# Evaluating efficacy of laser-assisted new attachment procedure and adjunctive low-level laser therapy in treating periodontitis: A single-blind randomized controlled clinical study

**DOI:** 10.1007/s10103-025-04457-0

**Published:** 2025-04-22

**Authors:** Fadime Kaya Dadas, Selin Genc Kocaayan, Mehmet Saglam, Omer Faruk Dadas, Serhat Koseoglu

**Affiliations:** 1 Katip Celebi University, Izmir, Turkey; 2 Ege University, Izmir, Turkey; 3 Istanbul Medeniyet University, Istanbul, Turkey

**Keywords:** Periodontitis, Laser, Root planing, Regeneration

## Abstract

**Purpose:**

This study aimed to evaluate the effectiveness of the laser-assisted new attachment procedure (LANAP) and low-level laser therapy (LLLT) on clinical, biochemical, and radiographic parameters when applied alongside scaling and root planing (SRP).

**Methods:**

The study was designed as a randomized controlled, single-blind, parallel trial involving 68 patients diagnosed with periodontitis. The participants were divided into three groups: Group 1: SRP (control), Group 2: LANAP, and Group 3: LLLT. Clinical measurements, gingival crevicular fluid (GCF) samples, and standard periapical radiographs were obtained pre-treatment and at one- and three-month follow-ups. In GCF, interleukin-1beta (IL-1β), interleukin-10 (IL-10), and vascular endothelial growth factor (VEGF) were analyzed.

**Results:**

In moderate (4–6 mm) and deep pockets (≥ 7 mm), laser-treated groups showed a significant reduction in pocket depth (PD) and clinical attachment level (CAL) compared to the control group. However, there were no statistically significant differences in biochemical markers between the groups. Group 2 demonstrated significant bone filling compared to the control group.

**Conclusion:**

In deep pockets, laser-treated groups provide additional benefits to SRP. The application of LLLT positively affected recession (REC).

**Trial registration number:** NCT04694222

**Date of registration:** 01/01/2021

**Supplementary Information:**

The online version contains supplementary material available at 10.1007/s10103-025-04457-0.

## Introduction

Periodontitis is an infectious disease that begins as gingivitis caused by microbial dental plaque (MDP) and can lead to inflammation, attachment loss, and bone loss if left untreated [1]. Nonsurgical periodontal treatment (NSPT) procedures, primarily consisting of scaling and root planing (SRP), are critical for eliminating MDP, preventing disease progression, and promoting periodontal health [2]. The effectiveness of SRP may be influenced by factors such as initial pocket depth, root concavity, furcation involvement, tooth type, and the presence of improper restorations [3]. These factors can hinder the complete elimination of MDP, potentially leading to the recurrence of periodontal disease due to bacteria invading the gingival tissue and dentin tubules [2]. To address this issue, systemic and local antibiotics and antiseptics [4], photodynamic therapy [5], and ozone treatment [6] are often used along with SRP. However, due to the side effects and disadvantages of these treatments, alternative methods are being investigated.

Currently, there is significant interest in researching various types of lasers to enhance the effectiveness of SRP in treating periodontitis. The neodymium: yttrium–aluminum-garnet (Nd:YAG) laser, with a wavelength of 1,064 nm, is a distinguished type of laser due to its high hemoglobin and melanin absorption, excellent hemostasis, and deep penetration into soft tissues [7]. The Nd:YAG laser also exhibits strong bactericidal and detoxification effects, along with biomodulation and anti-inflammatory properties [8].

The laser-assisted new attachment procedure (LANAP) is a method recently used with the Nd:YAG laser that has achieved promising results in treating periodontitis [9]. LANAP is a step-by-step procedure conducted in three stages [10]. In the first stage, the laser is used to remove the diseased epithelium, specifically targeting bacteria associated with periodontal disease, existing dental calculus, and thermolabile toxins that can be destroyed by heat [11]. In the second stage, root planing is performed to remove any remaining calculus, infected cementum, and granulation tissues. The third stage involves reapplying the laser to promote stable blood clot formation, preventing bacterial infiltration into the gingival sulcus and avoiding the epithelium from developing down into the sulcus [11, 12]. This procedure is the only laser treatment shown to contribute to periodontal regeneration by promoting the formation of new bone, cementum, and periodontal ligament, as well as improving periodontal clinical parameters [13, 14]. However, there are only a few randomized controlled clinical trials (RCCT) in the literature investigating the effectiveness of LANAP in treating periodontitis [9, 15, 16]. Furthermore, there are no RCCTs evaluating the biochemical and radiographic effects of LANAP. Therefore, this study is the first RCCT evaluating the clinical data along with the biochemical and radiographic outcomes of LANAP.

Another laser application that can enhance the effectiveness of periodontal treatment is low-level laser therapy (LLLT) [17]. LLLT has several positive effects on tissues, including reducing inflammation, promoting wound healing, enhancing immune system, managing pain, and stimulating the growth of new cells and tissues [17–19]. Recent in vitro studies using Nd:YAG low-level laser have shown that laser application not only facilitates periodontal regeneration but also significantly enhances osteoblast migration and Adenosine triphosphate production [20–22]. However, few studies have investigated the use of the Nd:YAG laser for LLLT along with SRP [23–26]. Moreover, in these studies, laser application has been observed to generally contribute to periodontal healing using a fiber optic tip. Notably, no studies have evaluated the additional effects of LLLT on SRP using a larger diameter (950-µm) R24 biomodulation handpiece. This study thus aims to assess the pure biomodulation effect of LLLT using this larger diameter Nd:YAG laser handpiece.

The hypothesis of this study is that the two laser procedures (LANAP and LLLT), which have demonstrated periodontal regenerative efficacy, will enhance periodontal healing when applied adjunctively to SRP. The originality of this research lies in being the first study to simultaneously evaluate the effects of both laser therapies (LANAP and LLLT) on SRP, while also conducting a comprehensive assessment of the relative efficacy of these two procedures through clinical, biochemical, and radiographic outcomes.

## Material and methods

### Study design, patient populations, and sample size calculation

This prospective, parallel-design, single-blind, RCCT was conducted per the Helsinki Declaration of 1975, as revised in 2013. The study protocol was approved by the Research Ethics Committee of Izmir Katip Celebi University of Medical Sciences (Version No. 20.09.2017). It was also registered with the Turkish Medicines and Medical Devices Agency (No. 10643207–511.06-E.251329) and the ClinicalTrials.gov database (Reference No. NCT04694222).

A total of 68 patients (40 males and 28 females, aged 26–68 years; mean age: 49.0 years) with periodontitis were recruited between October 2017 and March 2018 from the Department of Periodontics, Faculty of Dentistry, Izmir Katip Celebi University, Turkey. Eligible participants were informed about the study’s purpose and procedures and asked to provide written consent by signing a Volunteer Consent Form.

Based on a power analysis conducted using G*Power 3.1, a sample size of 20 individuals was determined to yield 98% power, assuming a 0.53 mm change in clinical attachment level (CAL) at a significance level of α = 0.05, as per a previous study [16].

### Determination of periodontal status and eligibility criteria

Patients were included based on the following criteria: diagnosis of periodontal stage II, III, or IV with grade B [27]; age between 25–70 years; systemically healthy; presence of at least 12 teeth; and having ≥ 4 teeth with pocket depth (PD) ≥ 5 mm, CAL ≥ 4 mm, and radiographic evidence of alveolar bone loss. Each quadrant had at least one tooth sampled. Exclusion criteria included smoking, pregnancy or lactation, recent antibiotic (last six months) or anti-inflammatory (last three months) use, systemic diseases, medication affecting periodontal condition, or restoration adjacent to sampled teeth.

### Clinical parameters

Periodontal status was assessed using clinical measurements, including PD, CAL, and recession (REC), evaluated at six sites (mesiobuccal, midbuccal, distobuccal, mesiopalatinal, midpalatinal, and distopalatinal) of each tooth. Additionally, the plaque index (PI) [28], gingival index (GI) [29], and bleeding on probing (BOP) [30] were measured at four sites (mesiobuccal, midbuccal, distobuccal, and midpalatinal) of each tooth.

Standardization was ensured by using a calibrated manual periodontal probe (PCP15-Hu-friedy, USA) for all patients. Measurements were conducted by a masked, calibrated researcher to maintain blinding. Intra-examiner agreement was assessed for PD (k = 0.96), and calibration was repeated in 10 patients with measurements taken one hour apart. Baseline measurements were repeated at the first and third months by the same examiner.

### Collection of samples

Following baseline clinical measurements, a second appointment was scheduled at least three days later for gingival crevicular fluid (GCF) collection. No periodontal procedures were performed before GCF collection to prevent influencing current periodontal status. The sampling was conducted during specific hours of the day, with saliva isolation achieved using sterile cotton rolls. Subsequently, supragingival plaque was removed, and tooth surfaces were lightly air-dried before paper strips (Periopaper®, OraFlow Inc., USA) were placed 1–2 mm into the gingival crevice for 30 s. Contaminated strips were excluded. The fluid volume was measured using a calibrated device (Periotron™ 8000 m; Oraflow Inc., USA), and strips from four quadrants were stored in coded polypropylene tubes (Eppendorf AG, Germany) at − 20 °C and subsequently at − 80 °C until analysis.

### Radiographic examination

After GCF sampling, standard periapical radiographs were taken from the region believed to have the deepest bone defect among the samples before SRP and at first and third months post-treatment. The parallel technique described in Khojastehpour et al.’s [31] study was utilized for this. The periapical radiographs thus obtained were transferred to a computer, and measurements were conducted using a specialized software program^1^ during evaluation. These measurements were performed following the methodology outlined by Eickholz et al. [32]. Changes in bone level were reported as a percentage.

### Intervention, periodontal treatment, randomization, and allocation concealment

In the second appointment, after the GCF collection was completed, patients were grouped randomly using opaque sealed envelopes. A masked investigator opened these envelopes.

Out of the 350 individuals assessed, 68 were included in the study. These patients were divided into the following three groups according to the aforementioned randomization:

- Group 1 (n = 24): receiving only scaling and root planing (control)
- Group 2 (n = 22): receiving scaling and LANAP (LANAP)
- Group 3 (n = 22): receiving scaling and root planing and LLLT (LLLT)

All treatments (SRP and laser procedures) were performed by the same investigator and completed within 24 h. After the initial samples were collected, patients in all groups received oral hygiene instructions, including the use of manual toothbrushes and dental floss or interproximal brushes. Before treatments, hand instruments were sharpened. Supragingival scaling was the first step for all groups.

For Group 1, root planing was conducted under local anesthesia^2^ using a combination of Gracey curettes^3^ and ultrasonic instruments^4^ until a smooth, clean surface was achieved for each tooth. Post-instrumentation, all supragingival surfaces were polished.

For Group 2, LANAP treatment followed scaling and polishing; it was performed in three stages:

1. In the first stage, the fiber-optic tip of the Nd:YAG laser (1,064 nm) 5 was placed parallel to the tooth’s long axis and inserted approximately 1 mm less than the PD. The fiber was slowly moved apically and laterally in a sweeping motion during the laser light emission. The laser was set at the following parameters: 3.0 W power, 180-µs (short pulse) pulse duration, and 20 Hz frequency, according to the preliminary study [14]. This ensure removal of the diseased epithelium toward the soft tissue wall of the periodontal pocket and significant hemostasis. Additionally, to prevent thermal damage and ensure standardization, the duration of the laser application was determined to be 10 s for each pocket.
2. In the second stage, full-mouth root planing was performed as in the control group.
3. In the third stage, an Nd:YAG laser fiber optic tip was applied at 4.0 W power, 320-µs (long pulse) pulse time, and 20 Hz frequency to achieve a stable fibrin clot. To avoid thermal damage to the gingiva during the laser application stages of LANAP treatment, physiological saline was applied with a blunt-tip 10 cc injector.

For Group 3, after SRP, LLLT was performed using the Nd:YAG laser (1,064 nm) with an R24 biomodulation handpiece tip (950-µm) at 1 W power, 10 Hz frequency, and 320-µs (long pulse) pulse duration. The spot size was 0,28 cm^2^ and energy density was calculated to be 106,15 J/cm^2^. The instrument was applied for 30 s per site (mesiobuccal, distobuccal, mesiolingual, and distolingual regions) at an average distance of 1 cm from the oral epithelial surface.

Patients in all groups received instructions on oral hygiene including the use of manual toothbrushes and dental floss or interproximal brushes. Mouth rinsing was discouraged post-treatment to avoid changes in results. After their periodontal treatments were completed, the patients were called for a follow-up two weeks later. Those with poor oral hygiene were motivated further; however, those who could not maintain optimal oral hygiene were dropped from the study. At the first and third months, patients were carefully re-evaluated to ensure they had performed effective plaque control.

### Biochemical analysis

GCF levels of interleukin- 1beta (IL- 1β), interleukin- 10 (IL- 10), and vascular endothelial growth factor (VEGF) were analyzed using enzyme-linked immunosorbent assay (ELISA) kits^6^. The total amounts of biochemical markers in four samples collected over 120 s were calculated.

To analyze the GCF samples, polypropylene tubes containing frozen paper strips were kept at room temperature for at least 20 min before 400 μl of phosphate buffer was added to each tube. The samples were kept at 4 °C overnight before analysis to ensure the passage of GCF into the phosphate buffer in the paper strips. Homogenization was achieved by vortexing the tubes for one minute, two hours before analysis. Diluted GCF samples were pipetted with polypropylene tips and analyzed for each biochemical mediator per the manufacturer's instructions. After the reaction was stopped with a solution, the absorbance was measured spectrophotometrically^7^ at a wavelength of 450 nm. Cytokine concentrations were adjusted based on GCF volume and expressed in pg/ml.

### Outcome variables

The primary outcome was the difference in the change of CAL between groups. Secondary outcome variables included differences among the groups in the following parameters: (i) PD, (ii) GI, (iii) PI, (iv) BOP, (v) mean levels of each biochemical parameter, and (vi) radiographic bone changes.

### Statistical analysis

Descriptive statistics of clinical, laboratory, and radiographic data were presented as mean, standard deviation, median, minimum, maximum, frequency, and percentage values. The normality assumption of the quantitative data was checked using the Shapiro–Wilk test.

For normally distributed variables, the repeated-measures analysis of variance (ANOVA) method was employed for intra-group comparisons, with Bonferroni correction applied for pairwise comparisons. Inter-group comparisons were conducted using the one-way ANOVA method along with Tukey’s HSD (honestly significant difference) multiple comparison test. Statistical analyses for normally distributed data were performed using the SPSS 25.0 package program (IMB, Armonk, NY).

Since moderate and deep pocket variables (PD, CAL, and REC) did not follow a normal distribution, the non-parametric Brunner-Langer method (F1-LD-F1 model) was used to examine the time-dependent variation in these variables. Analyses were performed using R 3.5.2 software. If the time-dependent change was dissimilar across groups (interaction p < 0.1), time comparisons within each group were conducted using the Brunner-Langer method (LD-F1 model) with Bonferroni correction. Furthermore, inter-group comparisons were performed using the Kruskal–Wallis method, with pairwise group comparisons conducted via the Dunn test. The level of significance was set at p < 0.05 for all analyses.

## Results

### Clinical findings

**Fig. 1:**
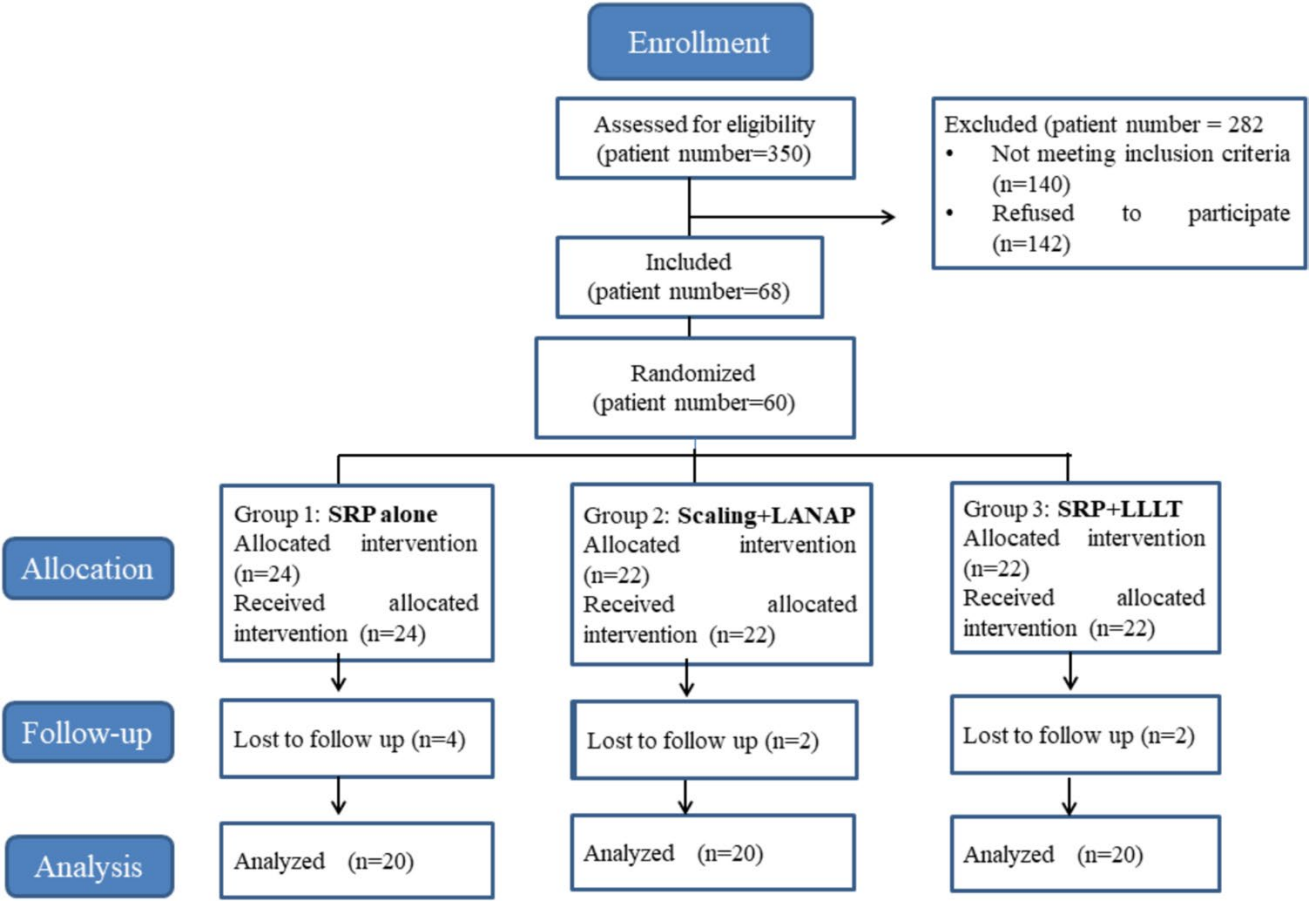
Flow Chart of the Study

Although the study initially enrolled 68 patients, it was completed with 60 participants, with each treatment group comprising 20 patients. Of the eight patients who discontinued participation, four failed to attend the first month’s appointment, and two missed the third month’s appointment. The remaining twos were excluded from the study for failing to comply with oral care recommendations (Fig. 1).

No adverse effects or complications related to SRP or laser treatments were reported throughout the study. The initial demographic characteristics of the patients are provided in Supplementary Table 1. At baseline, there were no statistically significant differences in the full-mouth periodontal pocket values of PI, GI, BOP, PD, and CAL across all groups (p > 0.05).

The PI, GI, and BOP values of all treated periodontal pockets (PD ≥ 4 mm) at each sampling time are presented in Supplementary Table 2. No statistically significant differences were observed between the groups at any time point (p > 0.05). Across all groups, significant reductions in PI, GI, and BOP values were noted at both the first and third months compared to baseline. However, in Group 3 (LLLT), a significant decrease in GI values was observed at the third month compared to the first month (p < 0.001).

Periodontal pockets were classified into two subgroups based on disease severity: moderate pockets (4–6 mm) and deep pockets (≥ 7 mm). A total of 2,378 moderate pockets were treated across all groups, with 759 in Group 1 (control), 859 in Group 2 (LANAP), and 723 in Group 3 (LLLT). Additionally, 569 deep pockets were identified, with 187 in Group 1, 219 in Group 2, and 163 in Group 3.

PD, CAL, and REC values for moderate pockets are shown in Table 1. In moderate pockets, a significant difference was observed in the inter-group comparisons of PD changes from baseline to the first month (p = 0.048). However, no significant differences were found in the pairwise comparisons (p > 0.05). Regarding CAL changes, a significant difference was observed only between Group 3 (LLLT) and Group 1 (control) (p = 0.007).

**Table 1:** PD, CAL, and REC parameters of all groups in moderate pockets over study periods

MODERATE POCKETS (4–6 mm)						Changes		
**Clinical parameters**	**Baseline (0)**	**First month (1)**		**Third month (3)**	***p v*alue**^a^	**0–1**	**0–3**	**1–3**
	Med (Min/Max)	Med (Min/Max)	Med (Min/Max)			Med (Min/Max)	Med (Min/Max)	Med (Min/Max)
**PD**								
Group 1 (Control)	5 (4/6)	3 (1/7) *	3 (1/7)* ^†^		** < 0.001**	2 (− 2/4)	2 (− 2/5)	0 (− 4/4)
Group 2 (LANAP)	5 (4/6) ^‡^	3 (1/7) *	3 (1/9) * ^†^		** < 0.001**	2 (− 2/4) ^‡^	2 (− 3/5) ^‡^	0 (− 4/4)
Group 3 (LLLT)	5 (4/6) ^§^	3 (1/8) *	3 (1/7)* ^† ‡^		** < 0.001**	2 (− 3/5)	2 (− 2/5) ^‡^	0 (− 3/5)
***p v*alue**^b^	**0.011**	0.184	**0.006**			**0.033**	**0.001**	0.207
**CAL**								
Group 1 (Control)	5 (4/11)	4 (1/11) *	4 (1/13) * ^†^		** < 0.001**	1 (− 4/4)	2 (− 4/5)	0 (− 4/5)
Group 2 (LANAP)	6 (4/12) ^‡^	4 (1/10) *	4 (1/11) * ^†^		** < 0.001**	1 (− 3/6)	2 (− 4/6)	0 (− 4/4)
Group 3 (LLLT)	5 (4/10) ^‡ §^	3 (1/9) * ^‡ §^	3 (1/8) * ^† ‡ §^		** < 0.001**	2 (− 4/5) ^‡ §^	2 (− 2/6) ^‡ §^	0 (− 3/5)
***p v*alue**^b^	** < 0.001**	** < 0.001**	** < 0.001**			**0.004**	** < 0.001**	**0.048**
**REC**								
Group 1 (Control)	0 (− 1/6)	1 (0/7) *	1 (0/7) *		** < 0.001**	0 (− 3/3)	0 (− 3/4)	0 (− 3/3)
Group 2 (LANAP)	1 (0/7)	1 (0/7) *	1 (0/8) *		** < 0.001**	0 (− 2/4)	0 (− 3/4)	0 (− 3/3)
Group 3 (LLLT)	0 (0/6) ^‡ §^	0 (0/6) * ^‡ §^	0 (0/6) * ^† ‡ §^		** < 0.001**	0 (− 2/3) ^§^	0 (− 2/3) ^‡ §^	0 (− 2/2)
***p v*alue**^b^	** < 0.001**	** < 0.001**	** < 0.001**			**0.003**	** < 0.001**	0.169

Med: Median, Min: Minimum, Max: Maximum, PD: Pocket depth, CAL: Clinical attachment level, REC: Recession.

^a^ indicates statistically significant difference for each group between baseline, the first month, and the third month, as analyzed using the Brunner-Langer method with Bonferroni correction (*p* < 0.05; * denotes a statistically significant difference compared to baseline, and ^†^ denotes a statistical significant difference compared to the first month).

^b^ refers to statistical analysis between groups at the same time point, as evaluated using the Kruskal–Wallis test with Dunn multiple comparison tests (*p* < 0.05; ^‡^ denotes a statistically significant difference according to Group 1, and ^§^ denotes a statistically significant difference compared to Group 2).

**Fig. 2:**
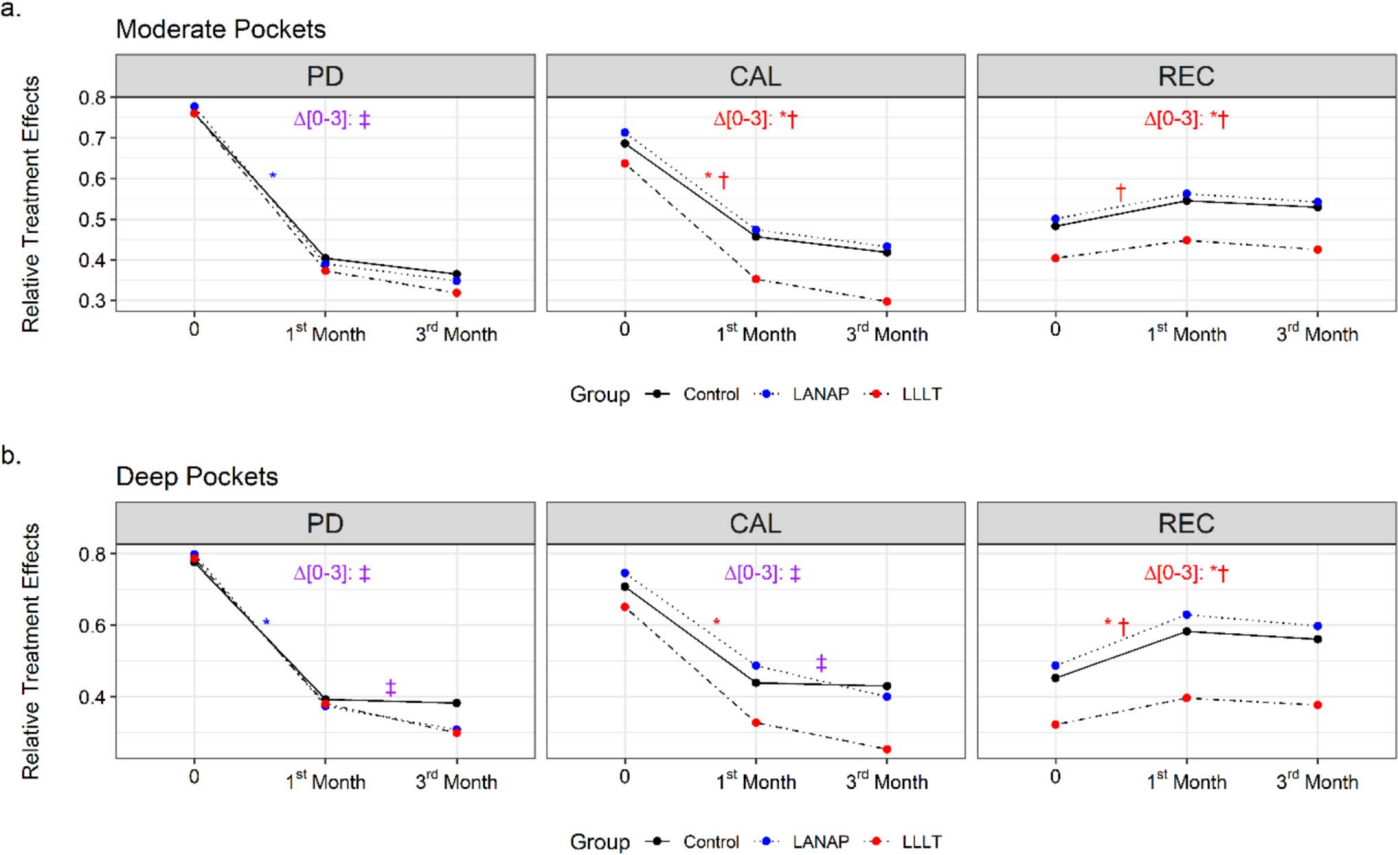
Time-dependent PD, CAL, and REC changes of all groups in **a.** moderate pockets and **b.** deep pockets

In the inter-group comparisons of PD changes from baseline to the third month, significant differences were found between the test groups (LANAP and LLLT) and Group 1 (control) (p = 0.002). Conversely, a significant difference in CAL was observed only between Group 3 (LLLT) and the other groups (p < 0.001). For REC changes, a significant difference was observed between Group 3 (LLLT) and the other groups (p < 0.001). As shown in Fig. 2a, REC was lower in Group 3 (LLLT), although the rate of change was minimal.

PD: Pocket Depth, CAL: Clinical Attachment Level, REC: Recession, 0: Baseline, Δ[0–3]: Changes in third month compared to baseline. The meanings of the symbols representing the statistically significant time-dependent changes between groups are as follows: * represents the difference of LLLT compared to the control. * represents the difference of LANAP compared to the control. ^†^ represents the difference of LLLT compared to LANAP. ^‡^ represents the difference of LANAP and LLLT compared to the control (Brunner-Langer method [F1-LD-F1 model]-R 3.5.2 software)

PD, CAL, and REC values of deep pockets are shown in Table 2. In deep pockets, intra-group evaluation revealed a significant reduction in PD in all groups at both the first and third months compared to baseline (p < 0.001). While the test groups showed a significant PD reduction at the third month compared to the first month, no significant reduction was observed in the control group. Statistically significant differences were found in the inter-group comparisons of PD changes (p < 0.001). Both from baseline to the first month and from the first to the third month, test groups exhibited a significantly greater PD reduction compared to the control group (p < 0.001). Consistent with this, test groups also demonstrated a statistically significant CAL gain compared to the control group (p < 0.001). A significant difference was observed in REC changes from baseline to the third month, particularly between Group 3 (LLLT) and the other groups. Group 3 (LLLT) exhibited a lesser degree of REC compared to the other groups (p < 0.001), consistent with the results observed in moderate pockets (Fig. 2b).

**Table 2:** PD, CAL, and REC parameters of all groups in deep pockets over study periods

DEEP POCKETS (≥ 7 mm)					Changes		
Clinical parameters	Baseline (0)	First month (1)	Third month (3)	p valuea	0–1	0–3	1–3
	Med (Min/Max)	Med (Min/Max)	Med (Min/Max)		Med (Min/Max)	Med (Min/Max)	Med (Min/Max)
**PD**							
Group 1 (Control)	7 (7/11)	5 (1/10) *	5 (1/9) *	** < 0.001**	3 (− 1/7)	3 (− 2/8)	0 (− 5/4)
Group 2 (LANAP)	7 (7/11)	5 (2/10) *	4 (1/10) * ^**†** ‡^	** < 0.001**	3 (− 3/7)^‡^	4 (− 2/9) ^‡^	0 (− 4/5) ^‡^
Group 3 (LLLT)	7 (7/11)	5 (1/10) *	3 (1/10) * ^**†** ‡^	** < 0.001**	3 (− 1/8)	4 (− 3/9) ^‡^	0 (− 6/6) ^‡^
***p v*alue**^b^	0.073	0.545	** < 0.001**		**0.032**	** < 0.001**	**0.002**
**CAL**							
Group 1 (Control)	8 (7/13)	6 (2/13) *	6 (2/12) *	** < 0.001**	2 (− 2/7)	2 (− 2/7)	0 (− 4/4)
Group 2 (LANAP)	9 (7/13) ^‡^	7 (2/14) *	6 (2/14) * ^**†**^	** < 0.001**	2 (− 3/7)	3 (− 3/8)^‡^	1 (− 5/5) ^‡^
Group 3 (LLLT)	8 (7/12) ^‡ §^	5 (2/12) * ^‡ §^	4 (2/12) * ^**†** ‡ §^	** < 0.001**	3 (− 3/7) ^‡^	4 (− 3/9) ^‡^	1 (− 5/6) ^‡^
***p v*alue**^b^	** < 0.001**	** < 0.001**	** < 0.001**		**0.012**	** < 0.001**	**0.001**
**REC**							
Group 1 (Control)	1 (− 2/7)	2 (0/8) *	1 (0/7) *	** < 0.001**	1 (− 1/3)	0 (− 1/5)	0 (− 2/3)
Group 2 (LANAP)	1 (0/5)	2 (0/7) *	2 (0/7) * ^**†**^	** < 0.001**	1 (− 2/5)	0.5 (− 2/4)	0 (− 4/2)
Group 3 (LLLT)	0 (0/3) ^‡ §^	0 (0/5) * ^‡ §^	0 (0/5) * ^‡ §^	** < 0.001**	0 (− 2/4) ^‡ §^	0 (− 1/3) ^‡ §^	0 (− 2/1)
***p v*alue**^b^	** < 0.001**	** < 0.001**	** < 0.001**		** < 0.001**	** < 0.001**	0.341

Med: Median, Min: Minimum, Max: Maximum, PD: Pocket depth, CAL: Clinical attachment level, REC: Recession.

^a^ indicates statistically significant difference for each group between baseline, first month, and third months, as analyzed by the Brunner-Langer method with Bonferroni correction (*p* < 0.05; * refers to statistically significant difference according to baseline, and ^**†**^ refer to statistical significant difference according to first month).

^**b**^ indicates statistical analysis between the groups in the same period, as analyzed by the Kruskal–Wallis and Dunn multiple comparison tests, (*p* < 0.05; ^‡^ refers to statistically significant difference according to Group 1, and ^§^ refers to statistically significant difference according to Group 2).

### Biochemical findings

The GCF volume decreased over time in all groups; however, no statistically significant differences were observed between the groups. Intra-group and inter-group comparisons of the total amounts of GCF IL- 1β, GCF IL- 10, and VEGF at baseline, the first month, and the third month are summarized in Table 3.

**Table 3:** GCF total amount of IL- 1β, IL- 10, and VEGF (mean ± SD) in all groups

**Biochemical parameters**	**Baseline** Mean ± SD	**First month** Mean ± SD	**Third month** Mean ± SD	***p* value**^a^
**IL- 1β (pg)/(60 s)**				
Group 1	32.64 ± 28.06	16.42 ± 5.36	16.50 ± 7.51	**0.028**
Group 2	23.04 ± 5.83	17.18 ± 7.19 *	16.69 ± 8.02 *	**0.011**
Group 3	24.80 ± 12.14	15.61 ± 8.76 *	14.09 ± 6.56 *	** < 0.001**
***p* value**^b^	0.075	0.054	0.356	
**IL- 10 (pg)/(60 s)**				
Group 1	0.73 ± 0.05	0.88 ± 0.23	0.91 ± 0.52	0.233
Group 2	0.77 ± 0.06	0.80 ± 0.05	0.78 ± 0.04	0.126
Group 3	0.79 ± 0.07	0.95 ± 0.39	0.82 ± 0.12	0.118
***p* value**^b^	**0.036**	0.065	0.581	
**VEGF (pg)/(60 s)**				
Group 1	15.65 ± 8.24	13.08 ± 3.81	11.86 ± 3.64	0.135
Group 2	17.63 ± 9.51	11.40 ± 2.92 *	9.99 ± 2.53 *	**0.004**
Group 3	17.57 ± 6.81	14.57 ± 6.10	12.71 ± 4.05 *	**0.020**
***p* value**^b^	0.403	0.297	0.115	

GCF: Gingival crevicular fluid, IL- 1β: Interleukin- 1β, IL- 10: Interleukin- 10, VEGF: Vascular endothelial growth factor, Min: Minimum, Max: Maximum, pg: picogram, s: second, SD: Standard deviation.

^a^ refers to statistically significant difference for each group between time intervals, using repeated measures ANOVA; Pairwise comparisons were performed by Bonferroni correction (*p* < 0.05; * refers to statistical significant difference according to baseline).

^b^ refers to statistically significant difference between groups in the same period, using one-way ANOVA, *p* < 0.05.

GCF IL- 1β levels showed a statistically significant reduction across all groups throughout the study, but no significant differences were found between the groups (p > 0.05). Similarly, the total amounts of GCF VEGF did not exhibit statistically significant differences between groups (p > 0.05).

For IL- 10, a statistically significant difference was observed between the groups at baseline (p < 0.036); however, no significant differences were noted at the first and third months (p > 0.05).

### Radiographic findings

Inter-group comparisons of the percentage changes in periodontal defects of all groups are presented in Table 4. At the first month, the highest bone filling was observed in Group 2 (LANAP), with a statistically significant difference compared to the control group (p = 0.021). By the third month, Group 2 (LANAP) continued to exhibit the highest bone filling; however, no statistically significant differences were found between the groups (p > 0.05).

**Table 4:** Percentage changes of radiographic bone fill in all groups

**Groups**	**∆B–1M** (% change) Mean ± SD	**∆B–3M** (% change) Mean ± SD
Group 1 (px)	0.71 ± 6.43	0.00 ± 7.24
Group 2 (px)	+ 4.68 ± 6.38 *	+ 6.44 ± 12.23
Group 3 (px)	+ 2.91 ± 4.90	+ 5.40 ± 3.42
***p* value**^a^	**0.021**	0.079

∆: Change, B: Baseline, 1 M: 1 month, 3 M: 3 months, px: pixel.

* refers to bone fill.

^a^ denotes statistically significant difference between groups in the same period, using one-way ANOVA and Tukey HSD multiple comparison test (*p* < 0.05; * refers to statistical significant difference according to Group 1).

## Discussion

To the best of our knowledge, this is the first RCCT to assess the clinical, biochemical, and radiographic effects of LANAP and LLLT besides SRP. This study’s primary finding is that both LANAP and LLLT significantly reduced PD and improved CAL, particularly in deep pockets with LLLT also demonstrating a notable positive impact on REC.

In the inter-group comparisons of changes in moderate pockets, it was found that the test groups showed a statistically significant reduction in PD at the third month compared to baseline. However, only Group 3 (LLLT) achieved a significant CAL gain at the third month relative to the control group. Additionally, Group 3 consistently exhibited the lowest REC in both moderate and deep pockets, suggesting healing via clinical attachment gain rather than gingival recession. LLLT’s ability to stimulate keratinocyte activity, accelerate wound healing, and enhance immune responses through fibroblast proliferation, matrix synthesis, and neovascularization [33, 34] likely contributed to this outcome. Furthermore, LLLT has been shown to improve tensile strength and gingival margin stability, aiding in REC prevention [35].

Conversely, the results in deep pockets showed slight differences. In intra-group evaluations, all groups demonstrated statistically significant reductions in PD compared to baseline at both the first and third months. However, by the third month, the test groups exhibited a statistically significant decrease in PD, while the control group did not. This finding suggests that when only SRP is performed in deep pockets, periodontopathogens may not be completely eliminated, leading to recolonization over subsequent months.

In inter-group comparisons for deep pockets, LLLT (applied to the oral epithelial surface outside the gingival sulcus) resulted in statistically significant reductions in PD and gains in CAL compared to the control group, similar to LANAP (applied internally within the gingival sulcus). To date, no study has specifically evaluated the effect of LLLT applied to periodontal pockets externally. Since LLLT is administered to the oral epithelial surface outside the gingival sulcus, it is unlikely to exert the same antimicrobial effects or ability to remove diseased tissue as LANAP [36, 37]. The benefits of LLLT in deep pockets attributed to its positive effects on tissue repair. Several studies have reported that LLLT enhances blood circulation, raises interleukin- 8 (IL- 8) levels, and modulates immune system cells, thereby improvin the immune response [38, 39]. In this study, LLLT was employed for biomodulation, and its contribution to periodontal healing likely stems from its enhancement of the host immune response.

While Group 2 (LANAP) provided reductions in PD, it did not yield a significant difference in CAL gain in moderate pockets. However, it did result in statistically significant CAL gain and PD reduction in deep pockets, likely due to the additional contributions of LANAP. Katuri et al. [16] stated that in deep pockets, LANAP provides greater reductions in PD and gains in CAL compared to SRP alone. Cobb et al. [40] examined the effects of the Nd:YAG laser on the root surface and subgingival flora. Microbiological studies involving LANAP have demonstrated its efficacy in reducing periodontal pathogens and promoting healing in difficult-to-access periodontal areas [36, 37, 41]. Additionally, LANAP has been reported to improve the de-epithelialization of diseased sulcular epithelium, potentially eliminating invasive bacteria [16]. Since access is more challenging in deep pockets than in moderate ones, LANAP’s ability to provide effective PD reduction in deep pockets is particularly relevant for clinical practice.

Biochemically, this study revealed significant reductions in the levels of IL- 1β across all groups. IL- 1β, a key mediator in the inflammatory cascade associated with periodontal disease, showed a consistent decline throughout the study period, with all groups demonstrating reductions by the third month. IL- 1β levels have been shown to decrease even after SRP alone due to resolution of inflammation [42]. Laser therapies applied in conjunction with SRP have shown positive effects in reducing IL- 1β levels [1]. Although some studies found no significant effects [25], the current study observed a consistent decrease over time, aligning with previous reports that highlighted positive effects. The lack of inter-group differences could be attributed to that SRP alone is sufficient to lower IL- 1β levels, as suggested by the literature [43]. In addition, the relatively long time frame of sample collection, which may have masked potential differences in early inflammatory responses. Although IL- 10 is generally known as an anti-inflammatory cytokine, its relationship with periodontal disease remains unclear. Literature findings on the effects of SRP and laser therapies on IL- 10 levels have been inconsistent, reporting decreases [44], no changes [45], or increases [46]. In the present study, no statistical differences in IL- 10 levels were observed between groups at any time point, consistent with studies reporting no significant changes. The VEGF levels, which play a crucial role in angiogenesis and tissue healing, decreased over time across all groups, which likely reflects the overall resolution of inflammation and the subsequent healing process. No significant differences were observed between groups regarding VEGF levels at any time point. Previous studies have reported reductions in GCF VEGF levels after SRP [47]. While studies on laser applications noted statistically significant increases in VEGF levels with both LLLT [48] and high-dose laser applications such as LANAP [49], these evaluations were conducted in the early healing period (24–72 h). Differences might have diminished in this study, where samples were collected at later stages.

The biochemical markers measured in this study were selected to provide insights into the molecular mechanisms underlying clinical outcomes. Together, the clinical and biochemical findings suggest that both LANAP and LLLT provide significant clinical benefits, potentially through modulation of inflammatory cytokines and promotion of tissue regeneration. The reduction of IL- 1β is consistent with the clinical improvements in PD and CAL, highlighting the role of inflammation in periodontal disease progression and healing. The involvement of VEGF in angiogenesis and bone regeneration is supported by the clinical improvements observed in bone fill, particularly in the LANAP group, where early regenerative effects were most pronounced.. The more significant effects of LANAP on bone healing and tissue attachment in deeper pockets may be attributed to its ability to target both diseased tissue removal and tissue regeneration, while the beneficial effects of LLLT on reducing gingival recession and promoting healing in moderate pockets may be attributed to its anti-inflammatory and biomodulatory effects.

Radiographically, although bone filling was observed across all test groups, only Group 2 (LANAP) demonstrated a statistically significant difference compared to the control group. While no large-scale studies have specifically evaluated LANAP’s effects on bone filling, case reports suggest that it can promote bone regeneration [50, 51]. This regenerative capacity is attributed to LANAP’s ability to form a physical barrier skin to a membrane that inhibits epithelial growth, stimulates the release of precursor cells from periodontal ligament and alveolar bone, and promotes stable fibrin clot formation. This clot is thought to enhance regeneration by directing healing from apical to coronal regions [50]. Conversely, LLLT has been shown to increase the release of growth factors [48], stimulate precursor cell production [17], and contribute to periodontal regeneration by reducing osteoclastic activity in periodontal tissues [52]. Further, LLLT contributes to early-stage bone formation by promoting osteoblast and fibroblast proliferation [53]. These mechanisms likely explain the bone filling observed in laser-treated groups and highlight the positive effects of LLLT and LANAP on bone healing [54, 55]. No statistically significant differences were noted between groups regarding changes in bone levels at both the first and third months.

This study has several limitations. First, the timing of biochemical sample collection might have affected the findings. Future studies should investigate the early healing effects of laser therapies combined with SRP on biochemical markers in periodontal tissues. Additionally, while this study monitored bone filling in the deepest defects, it did not standardize defect types. Further research focusing on specific bone defect types is warranted. Finally, bone assessments were performed linearly in this study. Volumetric changes should be evaluated using advanced and precise methods in future investigations.

## Conclusion

Both LANAP and LLLT have been shown to be effective adjunctive therapies to SRP in the management of periodontal disease. Additionally, both interventions contributed significantly to clinical outcomes, including the CAL gain and PD reduction, particularly in deep pockets. Notably, LLLT demonstrated a distinct advantage in minimizing gingival recession and promoting tissue healing, highlighting its potential as a valuable adjunctive treatment in periodontal therapy. Biochemically, a reduction in the inflammatory cytokine IL- 1β levels was observed in both treatment groups, indicating the ability of these therapies to control inflammation. However, no significant differences were found in VEGF and IL- 10 levels between the treatment groups, suggesting that these biomarkers did not vary significantly with treatment type. Additionally, both LANAP and LLLT treatments have demonstrated the potential for promoting bone gain. The significant advantages of these therapies suggest their potential to minimize the need for periodontal surgical interventions. However, further studies involving larger sample sizes and long-term outcome evaluations are necessary to confirm and expand upon these findings.

## Supplementary Information

Below is the link to the electronic supplementary material.

Supplementary file1 (DOCX 15 KB)

Supplementary file2 (DOCX 19 KB)

## Data Availability

No datasets were generated or analysed during the current study.

## Footnotes

1 Adobe Systems Incorporated, San Jose, CA, USA.

2 Maxicaine forte, VEM Ilac, Turkey.

3 Hu-Friedy, USA.

4 EMS minipiezon, France.

5 Fotona Fidelis AT, USA.

6 E-Bioscience, USA.

7 Multiskan GO, Thermo Fisher Scientific, MA, USA.
