# Supplementary material for: Evaluating efficacy of laser-assisted new attachment procedure and adjunctive low-level laser therapy in treating periodontitis: A single-blind randomized controlled clinical study
Source: Lasers Med Sci. 2025 Apr 22;40(1):208. doi: 10.1007/s10103-025-04457-0 (PMC12011649; doi:10.1007/s10103-025-04457-0)
Supplement: Supplementary file 1 — Supplementary file1 (DOCX 15 KB) [file 10103_2025_4457_MOESM1_ESM.docx]

|  | **Group 1** | **Group 2** | **Group 3** | ***p* value** |
| --- | --- | --- | --- | --- |
| **Age** *(years; mean ± SD)*  *(min/max)* | 49.25 ± 11.82  (28/68) | 49.40 ± 9.46  29/65 | 48.75 ± 11.77  26/69 |  |
| **Sex** *(male/female)* | (13/7) | (11/9) | (13/7) |  |
| **GI** | 1.82 ± 0.30 | 1.74 ± 0.29 | 1.70 ± 0.25 | 0.334 |
| **BOP** | 81.31 ± 21.79 | 73.27 ± 23.32 | 73.04 ± 21.12 | 0.409 |
| **PI** | 1.80 ± 0.41 | 1.91 ± 0.49 | 1.82 ± 0.49 | 0.079 |
| **PD** | 3.46 ± 0.96 | 3.69 ± 0.89 | 3.29 ± 0.69 | 0.262 |
| **CAL** | 4.19 ± 1.44 | 4.56 ± 1.32 | 3.78 ± 0.68 | 0.083 |

**TABLE 1.** Baseline demographic datas and clinical parameters

SD: Standard deviation, GI: Gingival index, PI: Plaque index, BOP: Bleeding on probing, PD: Probing depth, CAL: Clinical attachment level

*p* value refers to statistically significant difference between groups (*p<0.05)*, one-way analysis of variances (ANOVA)
