# Supplementary material for: Evaluating efficacy of laser-assisted new attachment procedure and adjunctive low-level laser therapy in treating periodontitis: A single-blind randomized controlled clinical study
Source: Lasers Med Sci. 2025 Apr 22;40(1):208. doi: 10.1007/s10103-025-04457-0 (PMC12011649; doi:10.1007/s10103-025-04457-0)
Supplement: Supplementary file 2 — Supplementary file2 (DOCX 19 KB) [file 10103_2025_4457_MOESM2_ESM.docx]

| **Index/Treatment** | **Baseline** | **1^st^ month** | **3^rd^ month** | ***P* value^1^** |
| --- | --- | --- | --- | --- |
| **PI** |  |  |  |  |
| Group 1 | 1.91 ± 0.60 | 0.35 ± 0.14 * | 0.30 ± 0.20 * | **<0.001** |
| Group 2 | 1.98 ± 0.47 | 0.35 ± 0.12 * | 0.20 ± 0.13 * | **<0.001** |
| Group 3 | 1.89 ± 0.49 | 0.35 ± 0.16 * | 0.27 ± 0.11 * | **<0.001** |
| ***p* value^2^** | 0.112 | 0.970 | 0.192 |  |
| **GI** |  |  |  |  |
| Group 1 | 1.88 ± 0.36 | 0.77 ± 0.19 * | 0.65 ± 0.32 * | **<0.001** |
| Group 2 | 1.80 ± 0.25 | 0.69 ± 0.17 * | 0.68 ± 0.20 * | **<0.001** |
| Group 3 | 1.78 ± 0.19 | 0.64 ± 0.17 * | 0.52 ± 0.15 * ^†^ | **<0.001** |
| ***p v*alue^2^** | 0.526 | 0.223 | 0.104 |  |
| **BOP** |  |  |  |  |
| Group 1 | 87.06 ± 20.91 | 20.60 ± 7.66 * | 18.10 ± 9.65 * | **<0.001** |
| Group 2 | 79.20 ± 20.23 | 18.98 ± 6.81 * | 18.88 ± 7.83 * | **<0.001** |
| Group 3 | 79.94 ± 15.27 | 15.62 ± 5.84 * | 13.13 ± 7.61 * | **<0.001** |
| ***p v*alue^2^** | 0.363 | 0.158 | 0.096 |  |

**TABLE 2.** PI, GI and % of sites with BOP mean score ± SD of treated teeth with PD ≥ 4 mm at baseline, and follow-up visits in all groups

SD: Standard deviation, GI: Gingival index, PI: Plaque index, BOP: Bleeding on Probing,

*p* Value**^1^** refers to statistically significant difference for each group compared to baseline (*p*<0.05), Repeated measures analysis of variances (ANOVA) (* refers to statistically significant difference for each groups between baseline and 1st month, ^†^ refers to statistical significant difference for each group between baseline and 3rd month)

*p* value^2^ refers to statistically significant difference between groups in the same period (*p<0.05)*, one-way analysis of variances (ANOVA) and Tukey HSD multiple comparison test
